# Molecular weight and viscosifying power of alginates produced by mutant strains of *Azotobacter vinelandii* under microaerophilic conditions

**DOI:** 10.1016/j.btre.2020.e00436

**Published:** 2020-02-20

**Authors:** Andres García, Tania Castillo, Diego Ramos, Carlos L. Ahumada-Manuel, Cinthia Núñez, Enrique Galindo, Jochen Büchs, Carlos Peña

**Affiliations:** a Departamento de Ingeniería Celular y Biocatálisis, Instituto de Biotecnología, UNAM, Universidad Nacional Autónoma de México, Ave. Universidad 2001, Col. Chamilpa, Cuernavaca, 62210, Morelos, México; b Departamento de Microbiología Molecular, Instituto de Biotecnología, UNAM, Universidad Nacional Autónoma de México, Ave. Universidad 2001, Col. Chamilpa, Cuernavaca, 62210, Morelos, México; c AVT – Chair of Biochemical Engineering, RWTH Aachen University,NGP2, Forckenbeckstraße 51, D-52074 Aachen, Germany

**Keywords:** Alginate, Weight-average molecular weight, Viscosifying power, Microaerophilic conditions

## Abstract

Alginates are polysaccharides that are of interest in various industrial applications. This is due to the viscosifying properties of alginates, which depends on the weight-average molecular weight. The aim of the present study was to evaluate the changes in alginate quality, in terms of the viscosifying power and weight-average molecular weight of the polymer produced by *Azotobacter vinelandii* mutant strains in shake flasks under microaerophilic conditions. In cultures developed at oxygen transfer rate (OTR) values close to 5 mmol L^−1^ h^−1^, the highest viscosifying power (1.75 L g^−1^) and weight-average molecular weight (3112 ± 150 kDa) were achieved in cultures performed with the AT9 strain. These values were higher than those obtained for the alginates produced by the parental strain ATCC 9046 grown under similar OTR conditions. In contrast, the alginate produced by the GG9 and OPAlgU + exhibited a very low weight-average molecular weight and therefore a poor viscosifying power. Our results have shown that by the cultivation of AT9 strain under microaerophilic conditions it is possible to obtain a polymer having a high weight-average molecular weight and excellent viscosifying capacity. Therefore, it could be a viable strategy for producing alginates for industrial applications.

## Highlights

• The highest molecular weight of alginate (3112 kDa) was obtained with the strain AT9.
• Culture broths of high viscosifying power were obtained using the AT9 mutant strain.
• RQ value is related with the synthesis of alginate and P3HB.

## 1 Introduction

Alginates are biopolymers containing β-d-mannuronic acid (M) and α-l-guluronic (G) acid monomers that are produced by bacterial species such as *Azotobacter vinelandii.* These polymers have gel-forming and viscosifying properties, besides, they are characterized by their non-antigenicity, biocompatibility and biodegradability [1,2]. The use of alginates has increased in areas such as tissue engineering, drug delivery and other biomedical applications. [1,[3], [4], [5]].

Both the alginate composition (MG/ratio) and its weight-average molecular weight influence the functional properties of the polymer [2,6]. In this regard, the viscosity and gelling properties, including the pre-gel solution viscosity and post-gelling stiffness, of the alginate are determined by its weight-average molecular weight [1]. Therefore, it is suitable to obtain polymers with an appropriate molecular weight for specific commercial applications, either as viscosifying or gelling agents.

It is possible to generate alginates having specific weight-average molecular weights by manipulating the culture conditions during the fermentation process ([7,8]; [4]). There are several studies in the literature which have demonstrated that the weight-average molecular weight and acetylation degree of alginates produced by wild-type *A. vinelandii* ATCC 9046 can be modified by the dissolved oxygen tension (DOT) and oxygen transfer rate (OTR). A full review can be found in Flores et al., [1] and Díaz-Barrera et al., [4]. For example, it has been shown that the weight-average molecular weight increases in cultures developed at low aeration conditions or low DOT (1 %) [1]. It was reported that in cultures developed at 700 rpm, at low DOT (3 %), the weight-average molecular weight increases, compared to cultures conducted at high DOTs (5 %) [9]. On the other hand, studies by Lozano et al., [10] have shown that under oxygen-limited and non oxygen-limited conditions, alginate production and its molecular mass are linked to the OTR_max_, independently of the DOT of the culture [10]. In this line, more recently Gómez-Pazarín et al. [11] found that, in cultures grown at low OTR_max_ (2.2 mmol L^−1^ h^−1^) the viscosifying capacity of alginate (relationship between viscosity and concentration) increased, with respect to that obtained in cultures with high OTR_max_ (5.5 mmol L^−1^ h^−1^) and this behavior was related with a high molecular mass of the polymer (2240 kDa).

Molecular strategies for the design of mutant strains of *A. vinelandii* have proven to be a very useful tool for alginate production with a high yield and specific chemical characteristics. Mutants as OPAlgU+ and GG9, among others, have shown to be potential sources for the production of alginate with a high molecular mass in shake flasks and bioreactor [12,13]. For example, strain GG9, an *A. vinelandii* mutant derivative of strain AEIV (also named E), which has the *mucG* gene inactivated (coding for a negative regulator of polymerization of alginates; [12]), has shown an alginate production increase of about 1.6-fold and a 2-fold increase at least of its mean molecular mass, with respect to the parental strain (AEIV). More recently, a mutant derivative of strain ATCC9046, named AT9, having the same mutation was designed.

In a more recent study, Castillo et al. [13] found that the *A. vinelandii* complemented strain OPAlgU+, in which alginate production was restored, showed a specific oxygen consumption rate (*q_O2_*) and alginate production very similar to that of strain ATCC9046. It is important to point out that in those studies, the strains were grown under diazotrophic and high aeration conditions (OTR_max_ = 30 mmol L^−1^ h^−1^), where alginate synthesis is not best.

In spite of the potential for the production of alginates having high weight-average molecular weight reported for these strains; the studies mentioned were carried out under culture conditions (nitrogen fixation and high oxygen transfer) that are not the best to promote the synthesis of high molecular weight alginate. Therefore, the aim of this study was to evaluate the viscosifying power and weight-average molecular weight of alginates synthesized by *A. vinelandii* mutant strains under microaerophilic conditions and no-nitrogen fixation.

## 2 Material and methods

### 2.1 Strains

The *A. vinelandii* strains used in this work were the wild type ATCC 9046, and its derivative mutant AT9 (*mucG::*mTn5; Sp^r^), the GG9 strain (*mucG::*mTn5) derived from the AEIV strain [12] and strain OPAlgU + which is an algU + complemented strain derived from the OP (*algU*^−^) strain [13]. In order to construct an ATCC 9046 derivative carrying the *mucG::*miniTn*5* insertion, this strain was transformed with 5 μg of chromosomal DNA from mutant GG9, according to previous reported [12]. Transformants were selected on BS media containing spectinomycin. Two representative mutants were confirmed, by PCR amplification of the *mucG locus*, to carry the *mucG*::miniTn*5* mutation (data not shown). The resulting strain was named AT9.

### 2.2 Culture medium

All strains were grown in modified Burk’s medium as previously described Gómez-Pazarín et al. [11]. The initial pH was adjusted to 7.2 using NaOH (2 N) before autoclaving. For maintenance of the AT9 and GG9 strains, 50 μg mL^−1^ of spectinomycin was used. In the case of OPAlgU + mutant strain, 2 μg mL^−1^ of kanamycin were added.

### 2.3 Culture systems

Cultures of the *A. vinelandii* strains were conducted in shake flasks of 250 mL with 50 mL of filling volume. Cultivations were maintained at 29 °C with a shaking frequency of 200 rpm in an orbital incubator with a shaking diameter of 25 mm. Before inoculation, protein was quantified in the inoculum by the Lowry´s method in order to adjust the inoculum volume to get an initial concentration of protein in the range of 0.06–0.08 g L^−1^, equivalent to 0.15–0.2 g L^−1^ of dry weight biomass, considering a protein content of 40 %. Evaluation of the OTR and the respiratory quotient (RQ) were determined by a respiration activity monitoring system (RAMOS), described by Anderlei et al. [14]. In addition, cultures were developed in a number of parallel flasks and submitted to off-line analytical measurements. All experiments were carried out at least in triplicate and results presented are the averages of the independent runs.

### 2.4 Analytical methods

#### 2.4.1 Cell growth and sucrose concentration assessments

Microbial growth was evaluated through protein measurements using the Lowry method, as described by García et al. [15]. Sucrose was evaluated using dinitrosalysilic acid reagent (DNS), as detailed by García et al. [15].

#### 2.4.2 Poly (3-hydrohybutyrate) (P3HB) and alginate quantification

P3HB content was quantified by HPLC after its conversion into crotonic acid, as it was previously described by Castillo et al. [16]. Alginate was measured by dry weight measurements using the method reported by Castillo et al. [16].

#### 2.4.3 Viscosity measurement and analysis of the weight-average molecular weight

Viscosity of culture broths was measured using a cone/plate rheometer as described by Peña et al. [17]. The alginate molecular weight distribution was estimated by gel permeation chromatography (GPC) with a serial set of Ultrahydrogel columns (UG 500 and Linear Waters), using a HPLC system with a differential refractometer detector (Waters, 410). Elution was performed with 0.1 M NaNO3 at 35 °C at a flow rate of 0.9 ml min^−1^. The detector signal was processed with a PC compatible software (Empower GPC, Waters). Calibration of the columns were performed via a standard calibration method using as molecular weight standards, the standard pullulans within a range of 58–2350 kDa. For this purpose, the standard kit P-82 (58–1600 kDa) and P2500 (2350 kDa) from Shodex.Co (Japan) were used. Alginate solutions were filtered through 0.22 μm Millipore membranes to remove microgels [9].

#### 2.4.4 Specific oxygen uptake rate determination

Equations used for the determination of specific oxygen uptake rates and respiratory quotient were the following:

(1) $$q_{O2}=\frac{OTR}{X}$$

(2) $$\text{R}\text{Q}=\frac{CTR}{OTR}$$

Where X_0_ (g L^−1^) was the initial protein concentration, *q_O2_* (mmol g^-1^  h^−1^) the specific oxygen uptake rate, RQ (-) is the respiratory quotient, CTR (mmol L^-1^  h^−1^) is the carbon dioxide transfer rate and OTR (mmol L^-1^  h^−1^) is the oxygen transfer rate.

The growth rate (μ) was calculated as described by Eq. 3

(3) $$\frac{dX}{dt}={Xoe}^{\mu t}$$

## 3 Results and discussion

### 3.1 Oxygen transfer rates in cultures of strains ATCC 9046, GG9, OPAlgU + and AT9

Considering the high mean molecular mass that the absence of MucG conferred to the alginate produced by mutant GG9, we wanted to explore this effect in the background of strain ATCC 9046, which is a natural alginate over-producing strain of *A. vinelandii*. For this purpose, we constructed, as described in Materials and methods, strain AT9, an ATCC 9046 derivative carrying a *mucG*::miniTn*5* insertion. In addition to the MucG deficient strains AT9 and GG9, the OPAlgU + strain was also included in this study.

**Fig. 1:**
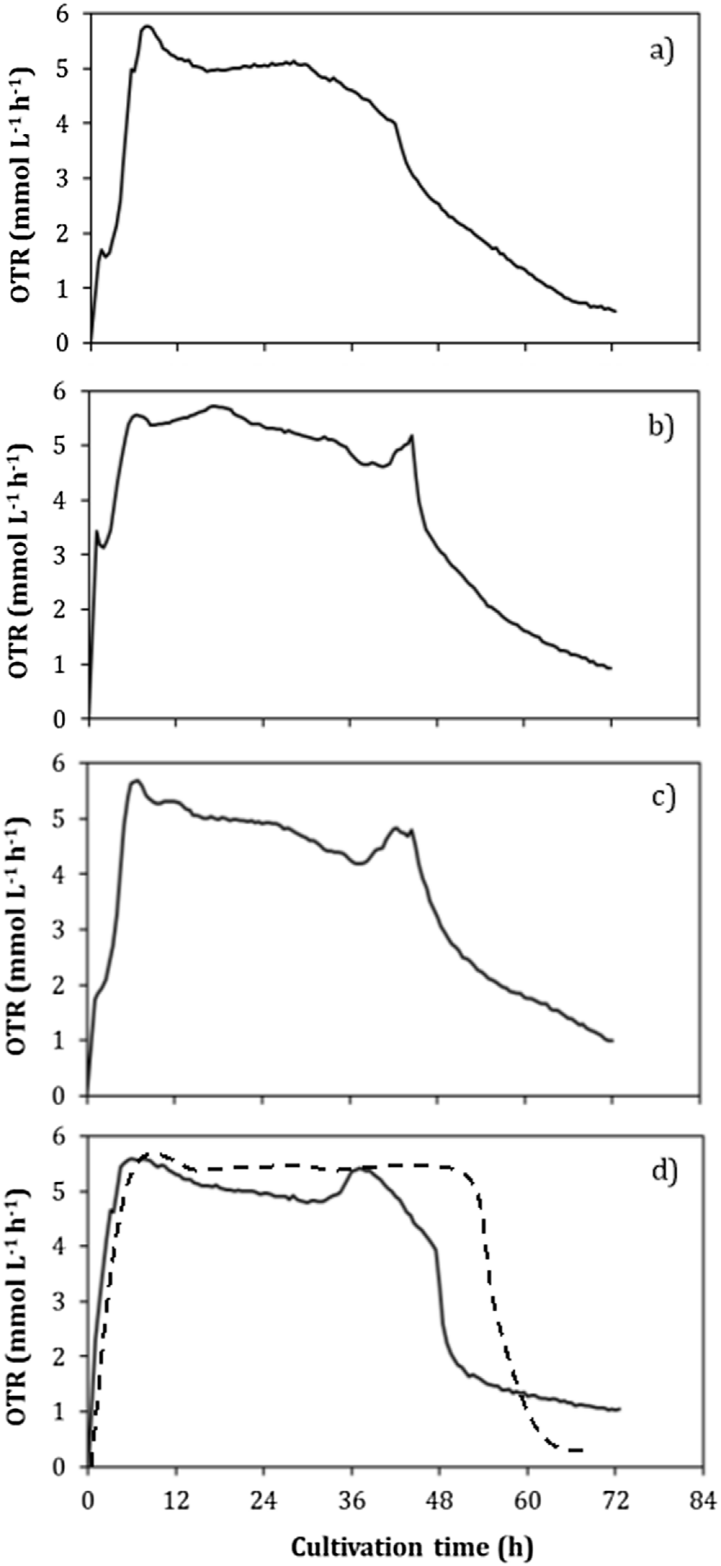
Evolution of the oxygen transfer rate of *A. vinelandii* strains, (a) ATCC 9046, (b) AT9, (c) GG9, and (d) OPAlgU+, developed in 250 mL shake flask cultures with a filling volume of 50 mL. The black dashed line represents the OPAlgU + strain grown with 4.5 g L^−1^ of yeast extract.

The oxygen transfer rate (OTR) evolution of the cultivations of strains ATCC 9046, AT9, GG9 and OPAlgU + are shown in Fig. 1. The maximum OTR (OTR_max_) value was reached after 5 h of cultivation and this remained practically constant until 30 h for the all strains evaluated. Specifically, the OTR_max_ reached values of 4.8 ± 0.28, 5.23 ± 0.18, 4.84 ± 0.33 and 5.24 ± 0.29 mmol L^−1^ h^−1^ for ATCC 9046, OPAlgU+, GG9 and AT9, respectively. After 30 h of cultivation, a straight increase in the OTR was observed in the cultures with the OPAlgU+, GG9 and AT9 strains. This increase could be related with changes in the metabolism of bacteria, from a condition of no-nitrogen fixation to a condition of nitrogen-fixation. It is known that when *A. vinelandii* is grown under diazotrophic conditions, a high respiration rate is induced by the respiratory protection of the nitrogenase [18]. In order to prove this hypothesis, cultures with the OPAlgU + strain with an excess of fixed nitrogen (4.5 g L^−1^ of yeast extract) were conducted. Analyzing the OTR profiles (Fig. 1d, represented by the dashed line), the peak after 30 h was not presented, suggesting that the diauxic growth behavior could be related to nitrogen exhaustion during the first stage, followed by diazotrophic growth. On the other hand, the highest values of maximum protein (X_max_ =1.15 ± 0.04 g L^−1^) and specific growth rate (μ = 0.10 ± 0.021 h^−1^) were achieved using the GG9 strain (Table 1). These values were significantly different from those obtained using the of OPAlgU + and AT9 strains, which only produced 0.80 ± 0.015 and 0.90 ± 0.017.

**Table 1:** Kinetic parameters of *A. vinelandii* strains cultured in 250 mL shake flasks. Experiments were carried out at least in triplicate and the results presented are the averages of the independent runs.

Strain	OTR_max_ (mmol L^−1^ h^-1^)	*q_O2_* (mmol O_2_ g_prot_^−1^ h^−1^)	RQ (-)	μ (h^−1^)	Maximal alginate (g L^−1^)	*qAlg* (g g^−1^ h^−1^)	P3HB (%)
ATCC9046	4.8 ± 0.28	13.6 ± 0.4	1.10 ± 0.08	0.09 ± 0.02	3.6 ± 0.6	0.050	58 ± 2.5
AT9	5.2 ± 0.29	16.4 ± 0.2	1.05 ± 0.05	0.09 ± 0.017	3.8 ± 0.2	0.052	39 ± 2.2
GG9	4.8 ± 0.33	12.9 ± 0.3	1.13 ± 0.05	0.10 ± 0.021	2.6 ± 0.5	0.037	60 ± 2.7
OPAlgU^+^	5.2 ± 0.18	15.2 ± 0.25	1.06 ± 0.05	0.08 ± 0.015	2.8 ± 0.2	0.046	61 ± 1.5

### 3.2 Respiration parameters

**Fig. 2:**
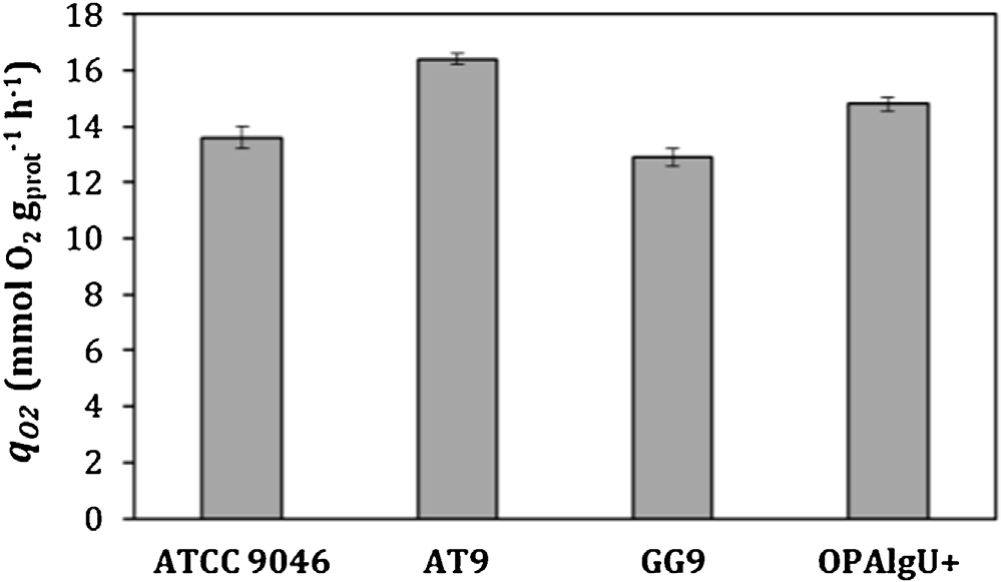
Specific oxygen consumption rates of *A. vinelandii* strains, ATCC 9046, AT9, GG9 and OPAlgU + developed in 250 mL shake flask cultures with a filling volume of 50 mL.

The evaluation of the respiration parameters, mainly the specific rate of oxygen consumption (*q_O2_*), is of great importance in order to understand the metabolism in each strain. The highest value of *q_O2_* was achieved in cultures with the AT9 strain (Fig. 2); it was 25 % higher than the *q_O2_* reached by GG9 and ATCC 9046 cultures, and 8 % higher with respect to the culture with the OPAlgU + strain (Table 1). These results contrast with those previously reported by Castillo et al., [13]. They described that, in cultures developed in shaken flask cultivations under oxygen limitation (microaerophilic conditions), the same used in the present study, the *q_O2_* values achieved by OPAlgU + and ATCC 9046 strains were higher than the values found in the present study. However, in those studies, *A. vinelandii* strains were grown under diazotrophic conditions. It is important to point out that the process of nitrogen fixation has a high energy requirement and it is even more important when *A. vinelandii* cells are grown under high aeration conditions due to the ‘respiratory protection’ mechanism of the nitrogenase system [19]. Based on this fact, our results suggest that under conditions of no-nitrogen fixation, such as those evaluated in the present study, the energy generated from the oxidation of the carbon source could be more efficiently distributed to produce biomass and alginate.

It is known that during alginate biosynthesis, one mole of ATP and one mole of GTP are consumed per monomeric unit, and two molecules of NADH are released [20]. Thus, it has been suggested that alginate biosynthesis results in a high oxygen consumption of the cells. This behavior was reflected by the *q_O2_* values and on the specific alginate production rate (*qAlg*) (Table 1). For example, the *qAlg* value obtained for the AT9 strain was higher compared to the GG9 and OPAlgU + strains, thus suggesting that in this strain, the carbon source and the oxygen are more efficiently utilized to produce alginate.

**Fig. 3:**
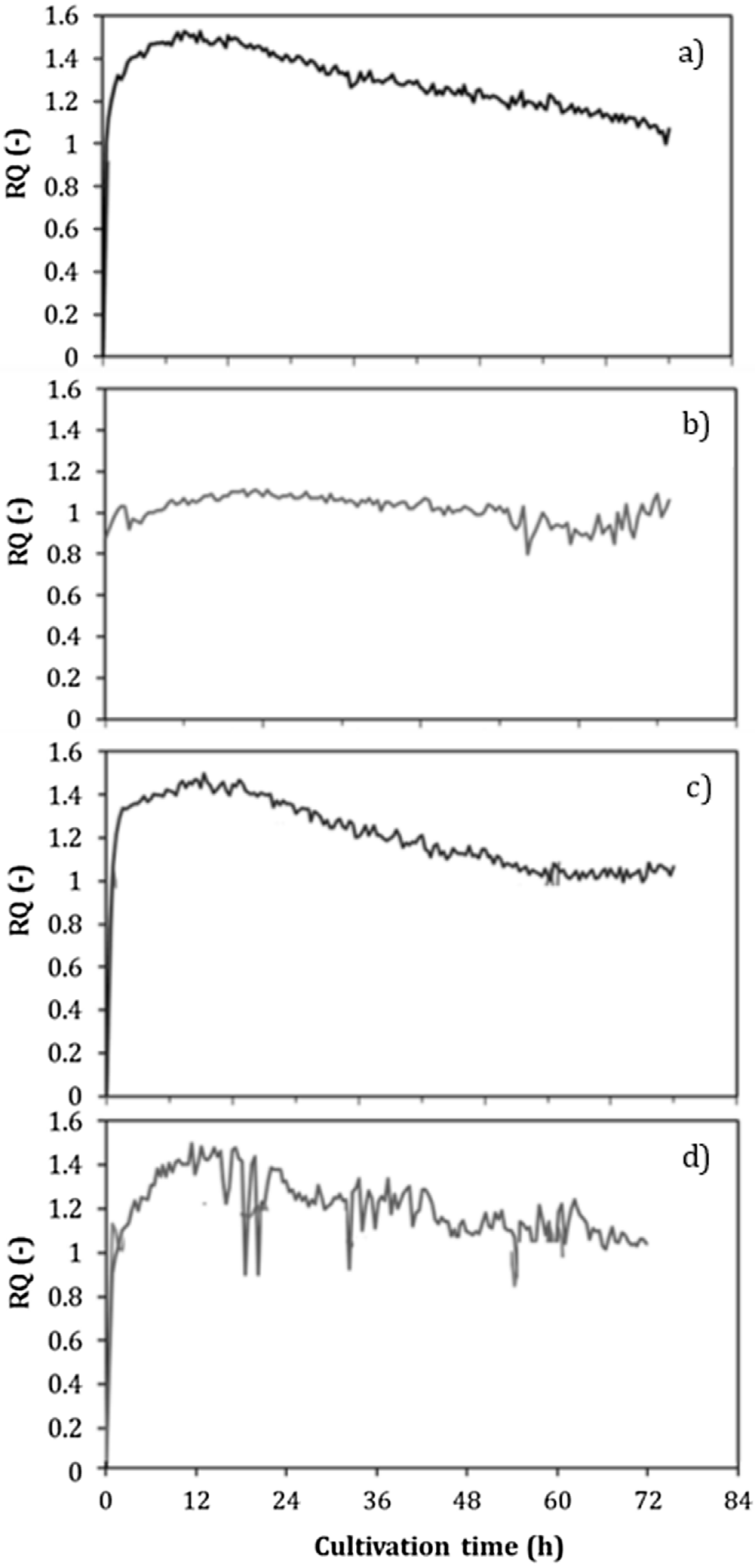
Evolution of respiratory coefficients (RQ) of *A. vinelandii* strains, (a) ATCC 9046, (b) AT9, (c) GG9 and (d) OPAlgU+, developed in 250 mL shake flask cultures with a filling volume of 50 mL.

**Fig. 4:**
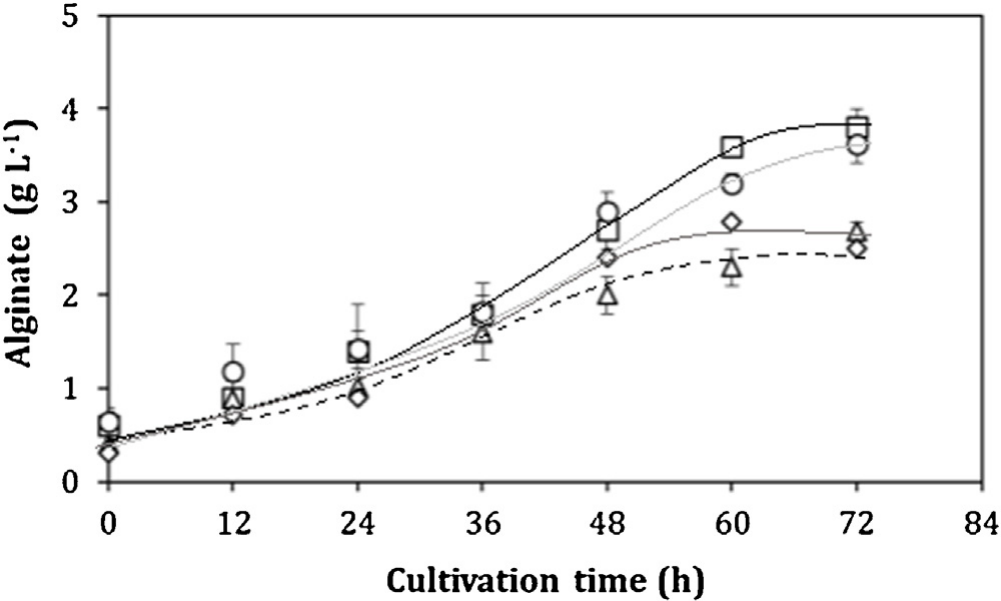
Alginate production kinetics of *A. vinelandii* strains, (○) ATCC 9046, (□) AT9, (△) GG9 and (◊) OPAlgU + developed in 250 mL shake flask cultures with a filling volume of 50 mL.

On the other hand, the respiratory quotient (RQ) is a very useful parameter for analyzing alginate and poly-3-hydroxybutyrate (P3HB) biosynthesis [7]. It is important to note that alginate and P3HB biosynthesis are closely related to energy metabolism and in both pathways there are points of generation and consumption of NAD(P)/NAD(P)H [20]. A low RQ value is associated with the highest conversion of the carbon source (sucrose) into alginate; whereas a high RQ value is typical of conditions where the carbon source is transformed to P3HB [7]. The RQ profiles recorded during the cultures by all strains are shown in Fig. 3. The average RQ was calculated during the stable plateau (between 5 and 25 h of cultivation). At values close to an OTR of 5 mmol L^−1^ h^−1^, the RQ was 8–10 % higher in the cultures with the ATCC 9046 and GG9 strains, with respect to the values for the cultures with the AT9 and OPAlgU + strains. These results suggest that the AT9 and OPAlgU + strains are channeling a higher amount of acetyl-CoA through the TCA cycle. This hypothesis is supported by the increase in 25 % of the *q_O2_* observed in cultures conducted with the AT9 strain, as well as in its RQ value of 1 (Fig. 3, Table 1). The energy of the carbon source oxidized under this condition would be used for cell growth and alginate production. As shown in Fig. 4, the highest alginate concentrations of 3.8 and 3.6 g L^−1^ were achieved by the AT9 and ATCC 9046 strains, respectively, and this value was associated with a lower RQ (1.05) for AT9 strain (Fig. 3). These differences were reflected in the *qAlg*, which was between 20 and 40 % higher with respect to the OPAlgU + and GG9 strains, respectively (Table 1).

**Fig. 5:**
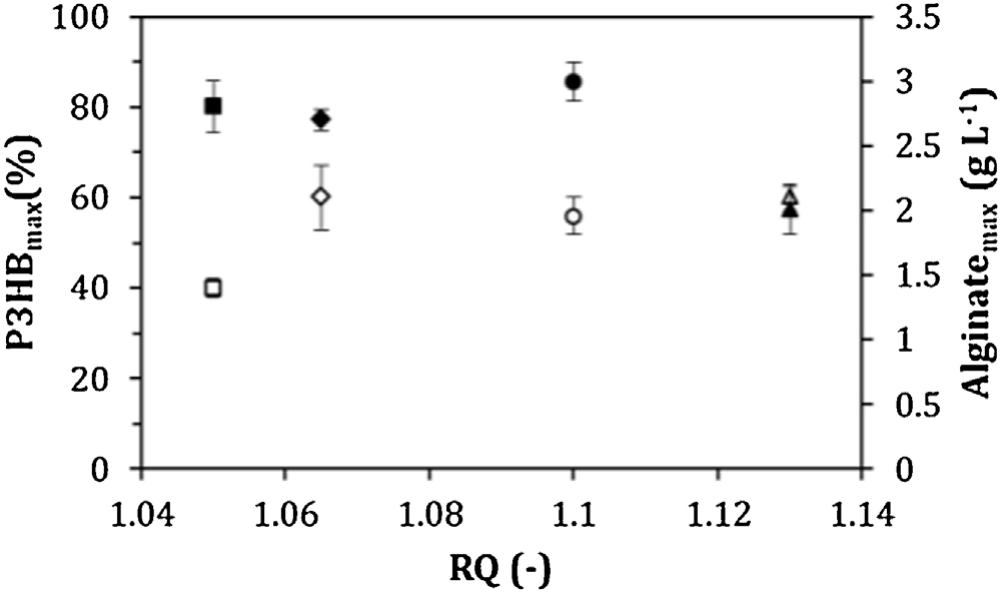
Correlation between RQ, alginate and P3HB production of *A. vinelandii* strains, (○) ATCC 9046, (□) AT9, (△) GG9 and (◊) OPAlgU + developed in 250 mL shake flask cultures with a filling volume of 50 mL. For alginate the symbols are closed, for P3HB the symbols are open.

Previous studies with the ATCC 9046 strain have shown that when the OTR, and consequently, the *q_O2_* increased, the carbon fluxes through the alginate biosynthetic pathway also increased [7,20,21]. In cultures using strains GG9 and ATCC 9046, the RQ values were higher than that obtained with the AT9 strain. As a consequence, the P3HB accumulation in GG9 and ATCC 9046 was up 60 % up. These values were similar with those previously reported for the cultures of the ATCC 9046 strain grown under similar growth conditions [7,11]. On the other hand, in the cultures conducted with the AT9 strain, P3HB accumulation was of 39 % (Table 1). According to these authors, when ATCC 9046 strain is grown at the lowest OTR, and therefore at the highest RQ, the carbon source is readily converted to P3HB. Fig. 5 shows that regardless of the OTR condition or the *A. vinelandii* mutant strains, low values of RQ (close to 1.05) were associated with a higher conversion of the carbon source into alginate; whereas high RQ values (close to 1.13) are related with the use of the carbon source for the P3HB biosynthesis.

### 3.3 Viscosifiying power of polymer solutions of the different strains

**Fig. 6:**
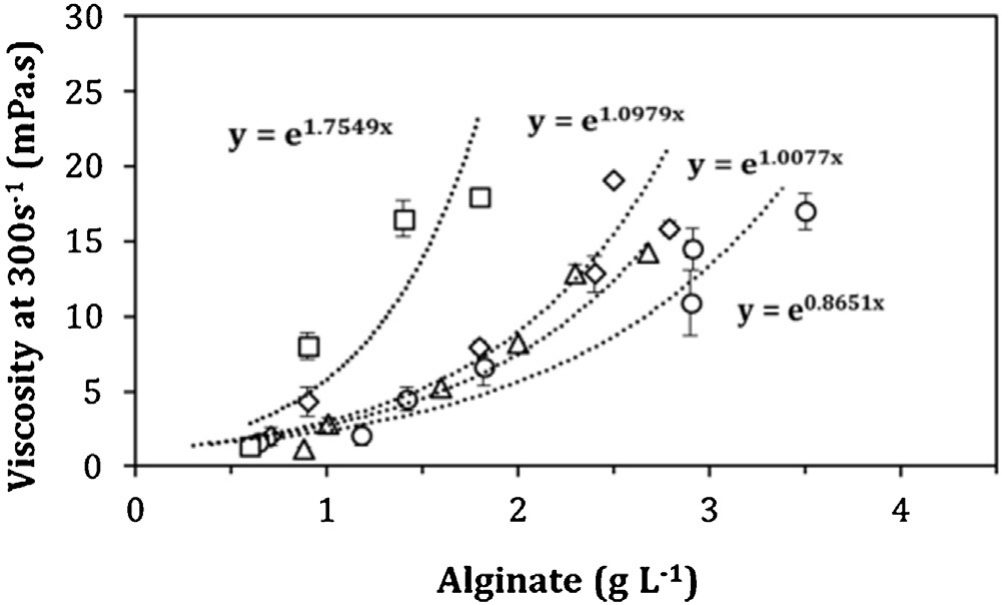
Viscosifiying power of the culture broth of *A. vinelandii* mutant strains, (○) ATCC 9046, (□) AT9, (△) GG9 and (◊) OPAlgU + developed in 250 mL shake flask cultures with a filling volume of 50 mL.

Alginates are commercially important because of their ability as viscosifier agents. Their quality depends on their viscosifying power, which can be defined as the exponent of the relationship between alginate concentration and the viscosity of the culture broth [7]. The apparent viscosity (measured at 300 s^−1^) reached by AT9 strain was higher (27 ± 1.8 mPas) with respect to that obtained with the wild type strain ATCC 9046 and than those determined for the GG9 and OPAlgU + strains (Table 2). Regarding the lower viscosities achieved in the cultures with the GG9 and OPAlgU + mutant strains, these are associated to the lower concentration of alginate produced by both strains. On the other hand, the lower apparent viscosities obtained for the culture broth of the ATCC 9046 strain are not related to the alginate concentration, because for this strain, the concentration of alginate was similar with respect to the AT9 strain. This was confirmed, when the viscosifying power of the culture broth of all strains was compared (Fig. 6). The highest value of viscosifying power (1.75) was obtained for the alginates produced using the AT9 strain; this value was twofold higher compared to the wild-type strain ATCC 9046 (0.86) (Table 2). It is interesting to note that the value of the viscosifying power for strain AT9 was 50 % higher to that reported previously for the ATCC 9046 strain cultured in shake flasks limited by oxygen [7,11]. In contrast, the viscosifying power reached by the GG9 and OPAlgU + strains were around 60 % with respect to those calculated for the AT9 strain grown under similar OTR condition (Fig. 6). One possible explanation for this behavior is the fact that the weight-average molecular weight of the alginate synthesized by the AT9 mutant strain is higher that of the wild type strain and the other mutant strains. Previous studies have reported that the viscosifying power is associated to a higher molecular weight and also to the acetylation degree of the polymer [7,10,11].

**Table 2:** Viscosifying power and molecular weight of alginates produced by *A. vinelandii* strains cultured in 250 mL shake flasks. Experiments were carried out at least in triplicate and the results presented are the averages of the independent runs.

Strain	OTR_max_ (mmol L^−1^ h^-1^)	*q_O2_* (mmol O_2_ g_prot_^−1^ h^−1^)	Molecular Weight (kDa)	Apparent viscosity (mPas)	Viscosifying power (L g^−1^)
ATCC9046	4.8 ± 0.28	13.6 ± 0.4	2170 ± 260	10.9 ± 1.5	0.86 ± 0.05
AT9	5.2 ± 0.29	16.4 ± 0.2	3112 ± 150	27 ± 1.8	1.75 ± 0.08
GG9	4.8 ± 0.33	12.9 ± 0.3	2300 ± 220	8.2 ± 0.7	1.00 ± 0.08
OPAlgU^+^	5.2 ± 0.18	15.2 ± 0.25	2100 ± 120	12.5 ± 0.5	1.09 ± 0.1

### 3.4 Weight average molecular weight of the alginates

**Fig. 7:**
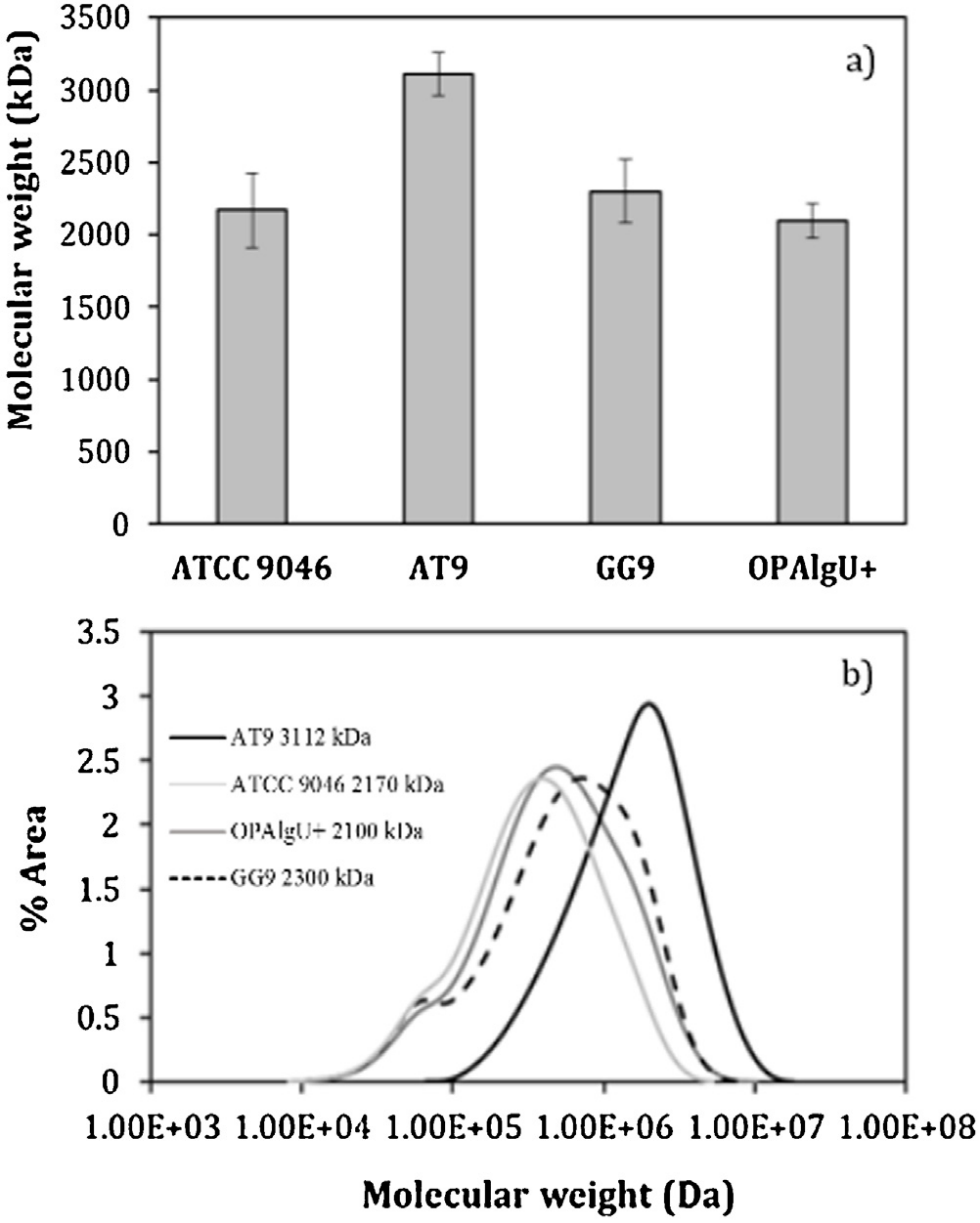
Weight-average molecular weight (a) and distribution of the weight-average molecular weight (b) of alginates produced by the *A. vinelandii* strains developed in 250 mL shake flask cultures with a filling volume of 50 mL.

As shown in Fig. 7, the highest weight-average molecular weight (3112 ± 150 kDa) was observed in the alginate isolated from the cultures developed with AT9 mutant strain at OTR_max_ value of 5.0 mmol L^−1^ h^−1^. This value was 30 % higher with respect to that obtained for the wild-type strain ATCC 9046 (2170 ± 260 kDa). This value of weight-average molecular weight was higher than that previously reported for this strain grown under similar OTR_max_ conditions. For example, Gómez-Pazarín et al., [11] reported a maximum weight-average molecular weight value of 1700 kDa; whereas Peña et al., [7] found an increase in the weight-average molecular weight of alginates from 1750 ± 50 to 1880 ± 40 kDa in cultures developed at an OTR of 6.0 and 2.6 mmol L^−1^ h^−1^, respectively. To our knowledge, the weight-average molecular weight of the alginate produced by AT9 strain is one of the highest reported so far, and also we confirmed that MucG inactivation results in the production of alginates with a high weight-average molecular weight.

In a previous work, it was reported that the MucG protein had a negative effect on the alginate molecular mass [12]. The culture was conducted under nitrogen-fixing conditions using strain AEIV, a wild type isolate producing moderate amounts of this polymer when compared to strain ATCC 9046. The high weight-average molecular weight of the alginate produced by strain AT9, a MucG-deficient, ATCC 9046 derivative mutant, confirmed the role of MucG as a protein negatively controlling the alginate chain length. As it is shown in Fig. 7a and Table 2, the maximum weight-average molecular weight of the alginates synthetized by GG9 and OPAlgU + strains, grown under microaerophilic conditions, were 2100 120 ± and 2300 ± 220 kDa, respectively. It must be emphasized that the value of the weight-average molecular weight obtained for the GG9 strain contrasts to the behavior reported for this strain grown under diazotrophic conditions, in which the alginate polymerization was lower (1451 ± 411 kDa) and appears to be unaffected by changes of the OTR values, due to the absence of MucG [12]. These data suggest that the molecular weight of the alginate produced by the GG9 strain could be modulated by the nitrogen-fixing conditions of the cell.

Fig. 7b shows the weight-average molecular weight distribution of alginates produced by the strains tested in this work grown under microaerophilic conditions. The weight-average molecular weight distribution of alginates produced by the AT9 strain was within the range of up to 100 and 10,000 kDa. In contrast, the weight-average molecular weight distribution of the alginate produced by the OPAlgU+, GG9 and ATCC 9046 strains varied between 10 and 10,000 kDa. The differences of the weight-average molecular weight distributions among the AT9 and the other strains suggest that in this strain there could be differences in the regulation of the polymerization and depolymerization of alginates in these strains. Previous works have described that the high weight-average molecular weight of alginates obtained at low dissolved oxygen, could be the result of a higher polymerase activity [1,22]. It is important to note that MucG is a multi-domain, inner membrane protein that has been proposed to prevent the accumulation of the second messenger c-di-GMP [12]. Therefore, in the absence of MucG, a higher c-di-GMP pool would enhance the polymerase activity of the Alg8-44 complex, by binding to the Alg44 PilZ domain, producing alginates of higher weight-average molecular weight. However, more studies are needed for a better understanding of the factors determining the molecular mass of the alginate produced by *A. vinelandii* and the molecular mechanism by which MucG controls this trait.

## 4 Conclusions

Our results have shown that the viscosifying power, as well as the weight-average molecular weight of the alginates produced are affected by the strains under study. It was possible to obtain alginates with a high weight-average molecular weight and cultures broths of high viscosity using the mutant AT9 under non-diazothrophic conditions. This finding will open up new possibilities for the implementation of alternative molecular strategies for the scaling-up of alginates with improved viscosifying.

## Declaration of Competing Interest

The authors declare that they have no competing interests.

## CRediT authorship contribution statement

**Andres García:** Investigation, Writing - original draft. **Tania Castillo:** Investigation, Conceptualization. **Diego Ramos:** Investigation. **Carlos L. Ahumada-Manuel:** Writing - review & editing. **Cinthia Núñez:** Writing - review & editing. **Enrique Galindo:** Writing - review & editing. **Jochen Büchs:** Writing - review & editing. **Carlos Peña:** Writing - review & editing, Supervision, Project administration, Funding acquisition.
